# The impact of precipitation on ANC service utilisation and healthcare access in Malawi

**DOI:** 10.1038/s41598-025-21645-8

**Published:** 2025-10-29

**Authors:** Rachel E. Murray-Watson, Margherita Molaro, Rebecca J. Murray-Watson, Sakshi Mohan, Bingling She, Tara Mangal, Joseph H. Collins, Sangeeta Bhatia, Eva Janoušková, Timothy B. Hallett

**Affiliations:** 1 MRC Centre for Global Infectious Disease Analysis, Jameel Institute, School of Public Health, Imperial College London, London, UK; 2 European Centre for Medium-Range Weather Forecasts, Reading, UK; 3 Centre for Health Economics, University of York, York, UK; 4 Institute for Global Health, University College London, London, UK

**Keywords:** Climate change, Population health, Extreme weather events, Climate disruptions, Healthcare systems, Antenatal care, Health care, Health policy, Health services, Climate-change impacts

## Abstract

Malawi is vulnerable to climate-related shocks, which are projected to worsen. Whilst some dimensions of this vulnerability have been characterised, little is known about healthcare sector resilience. Coupling facility-specific data on antenatal care (ANC) service provision in Malawi with gridded precipitation data from 2012-2024 we use linear regression analyses to characterise the historic relationship between precipitation and healthcare access. We estimate that precipitation negatively impacted ANC service utilisation in Malawi, with up to 1 in 20 appointments disrupted annually in some districts. Projecting further to 2060 indicates that, cumulatively, up to 250,000 pregnancies could be affected. Notably, if precipitation patterns from 1941 to 1953 had persisted into the 21st century, disruptions between 2012 and 2024 would be a hundred times less frequent, highlighting the significant influence of anthropogenic climate change on healthcare access. In a country already facing high maternal and neonatal mortality, such disruptions could further hinder access to care and worsen health outcomes. To mitigate this, interventions should focus on preserving or improving the physical accessibility of facilities, particularly through resilient transport services and road networks.

## Introduction

In the Intergovernmental Panel on Climate Change’s 2022 Report^1^, Malawi was identified as one of the most vulnerable countries to climate change. Whilst this vulnerability has been explored in terms of food security^2,3^, economic activity^4–6^, and some infectious diseases (e.g. malaria,^7^), little consideration has been given to the effect climate - and particularly, extreme weather events - has on the access to healthcare and health system functioning. Yet with many low-lying regions, unpaved roads, and poorly constructed buildings, Malawi’s health infrastructure is vulnerable to a variety of weather events, especially extreme precipitation, flooding, and landslides^7^.

Indeed, between 1991 and 2020, more than 3.5 million people were exposed to flooding in Malawi, with 935 confirmed deaths related to flooding, e.g. due to mudslides, disease, and injury^7^. In addition to these direct impacts on individual health, extreme precipitation events can severely disrupt access to and provision of medical care. In 2023, Cyclone Freddy affected patients’ access to 74 healthcare facilities, and hindered the operation of a further five facilities^8^, one of which was forced to close for 17 months^9^. The frequency of similar extreme weather events has been increasing in Malawi in recent decades, with more tropical cyclones recorded between 2012–2023 than occurred between 1946–2008^7^. Given that Malawi’s average temperature has already increased 1.1$^\circ \hbox {C}$ from 1950 to 2022 (from 21.2 to 22.3 $^\circ \hbox {C}$)^10^, such trends in extreme weather may be expected to persist as the climate continues to warm. Indeed, the World Bank posits that floods will be the most prominent climate-related threats Malawi faces^7^. However, since there is no systematic data collection on the impact of these and other weather-mediated disruptions on healthcare supply or access (such as road disruptions or a diminished healthcare workforce) the magnitude of their consequences for Malawi is unknown.

In other regions, some evidence linking healthcare access and adverse weather events has been found. Both the level of precipitation, the accessibility of a facility via paved roads - and the interaction between both - have been linked to reduced healthcare utilisation^11^, with even moderate precipitation events having negative consequences. Flooding cuts off access to healthcare^12^, and in regions such as Jakarta, Indonesia^13^ and Assam, India^14^, this exacerbates existing issues with healthcare supply. Fewer institutional deliveries occur in Mozambique’s rainy season compared to the dry season^15^, whilst flooding events have been linked to lower utilisation of antenatal care (ANC) services in parts of Bangladesh^12^.

There are limited such studies for Malawi: Cyclone Freddy was shown to reduce ANC utilisation in affected regions^16^, though there is no estimation of the effect of cyclones or precipitation beyond this specific event, nor of the future impact. Around 80% of the roads in Malawi are unpaved^17^, and thus are susceptible to heavy precipitation, providing a plausible mechanism of disruption. During Cyclone Freddy, roads in affected regions were rendered inaccessible due to flooding and landslides^18,19^, severely impinging on humanitarian responses. With the lack of transport to clinics already cited as a barrier to care among women seeking ANC services^20,21^, it is likely that unpaved roads’ vulnerability to precipitation - especially extreme precipitation - compounds the issue and drives down ANC utilisation even further^11,15^.

Here, we quantify the effects of precipitation on the utilisation of ANC services in Malawi. Compared to other healthcare services, ANC appointments are moderately time-constrained: definitionally they must precede the end of pregnancy. Therefore, disruptions to ANC services are comparatively more likely to cause missed appointments, rather than to delayed appointments (though, earlier in pregnancies, delays of a few weeks are possible). Additionally, given that future ANC service utilisation is primarily associated with demography, the demand for these services is simpler to project than that of, for example, infectious diseases. Importantly, attendance of ANC appointments is critical in reducing neonatal and maternal morbidity and mortality^22–25^, meaning that weather-mediated disruptions to these services could have severe health consequences. The utilisation of ANC services is a strong predictor of engagement with other maternity services, including institutional delivery and postnatal care. Consequently, disruptions in ANC services can serve as an indicator of broader healthcare service disruptions^26,27^.

Using facility-specific data on ANC service utilisation in Malawi^28^ coupled with climate data from 2011 to 2024^29^, we use regression analyses to characterise the historic relationship between precipitation and ANC service delivery. We then use spatially downscaled climate model projections of precipitation for the period 2025-2060^30^ to estimate the impact across three divergent Shared Socioeconomic Pathways (SSPs). We estimate that, between 2012 and 2024, utilisation of ANC services was adversely affected by precipitation in Malawi, with up to 1 in 20 annual appointments disrupted in some districts. Future projections broadly replicate these historic patterns: using projections on future pregnancies from the TLO, we find that a quarter of a million affected pregnancies between 2025 and 2060. Interestingly, if precipitation patterns from 1941-1953 had persisted into the early 21st century, there would be a hundred-fold fewer disruptions, indicating the consequential role of anthropogenic climate change in the provision of healthcare services. In a country with already-high maternal and neonatal mortality^31–33^, such disruptions could increase barriers to care and worsen health outcomes.

## Historical relationship between precipitation and ANC appointments

**Fig. 1:**
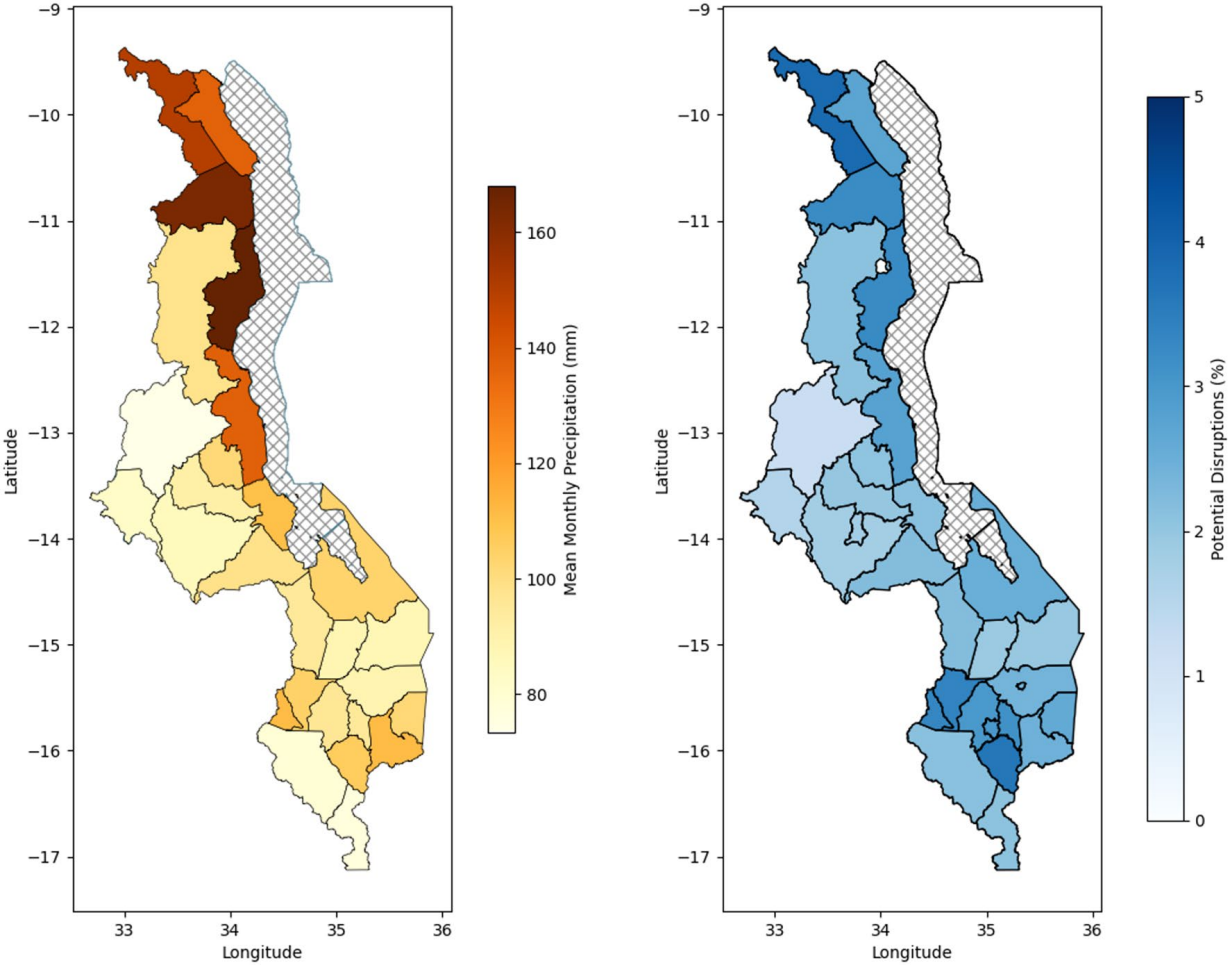
(**A**) Mean monthly cumulative rainfall (mm) per month for 2011 to 2024 by district. (**B**) Percent of ANC visits disrupted by precipitation by district between 2011 and 2024. Maps were created by the first author in Python using Shapely 2.1.1^36^.

For our outcome metric, we use the total number of ANC services delivered per clinic each month. We obtained these data from Malawi’s District Health Information Software (DHIS2;^28^) and used monthly records between January, 2011 and October, 2024 (see section “Methods”). For our precipitation variables, we used gridded reanalysis data for 2011–2025^29^, locating clinics to the appropriate grid square using a KDTree sorting algorithm^34^. From this we calculated several monthly precipitation metrics, informed by the Expert Team on Climate Change Detection and Indices (ETCCDI,^35^): the monthly maximum 5-day consecutive precipitation (Rx5day), the monthly cumulative precipitation (Fig. 1 A), and various lags in these metrics.

We used backwards stepwise selection to create two linear regression models to predict future ANC visits (“Analysis”, ^15^): one without any precipitation indices ($M_0$), and one including precipitation indices ($M_1$). In $M_0$, the setting of the facility (urban or rural), the owner of the facility (Government, Christian Health Associations of Malawi (CHAM), Non-Governmental Organisation (NGO), or Private), and its Euclidean distance to the next closest facility were significantly associated with counts of ANC appointments (Table 1, section “Analysis”). For the precipitatin indices in $M_1$, only the cumulative monthly precipitation was significant, though other metrics were included in the final, selected model.

**Fig. 2:**
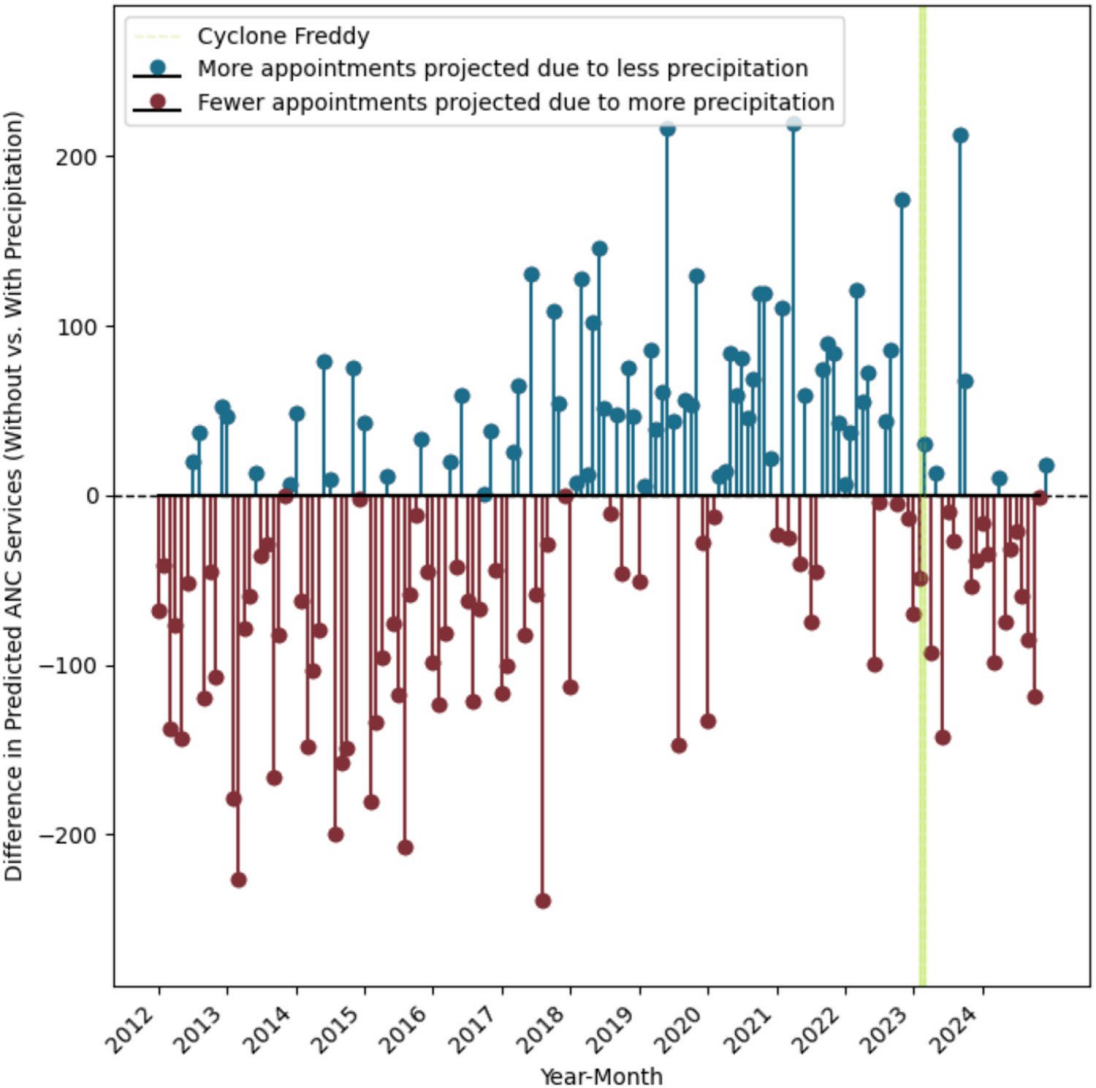
Monthly projected disruption of ANC service delivery across Malawi, calculated as the difference between models $M_0$ and $M_1$ (without and with precipitation indices respectively) for years 2011–2024. The green bar indicates months affected by Cyclone Freddy (February and March, 2023), where extreme rain fell in the southern part of Malawi and limited the operation of several healthcare facilities.

The difference in predictions between these two models gave us our expected change in ANC services used due to precipitation (Fig. 2, ^15^). Though the coefficient for the cumulative monthly precipitation was small (− 0.0004 ANC visits per facility per month per millimetre of monthly precipitation, Table 1), this translated into large effects with up to 4.7% of cases affected in some districts (mean across all districts of 2.5% 95% CI 2.25 – 2.73); Fig. 1B). This translates to an estimated 66,000 affected pregnancies over 13 years, assuming that one live birth equates to around 4.4 ANC appointments delivered (accounting for stillbirths;^37,38^).

**Table 1:** Regression results, including coefficients and p-values for various predictors, based on a models developed on data from 2011 to 2024.

Variable (unit)	$M_0$	p-value	$M_1$	p-value
Year	0.0021	0.0000*	0.0021	0.0000*
Month	0.0047	0.0317*	0.0048	0.0295*
Altitude (m)	0.00005	0.0511	0.00005	0.0596
Minimum distance to closest clinic (m)	3.0343	0.0000*	3.0455	0.0000*
Rural	Reference	–	Reference	–
Urban	1.3042	0.0000*	1.3039	0.0000*
Central East	Reference	–	Reference	–
Central West Zone	0.1277	0.0000*	0.1230	0.0000*
Northern Zone	– 0.5821	0.0000*	– 0.5623	0.0000*
South East Zone	0.1089	0.0001*	0.1036	0.0002*
South West Zone	0.0487	0.1132		
CHAM	Reference	–	Reference	–
Government	0.2592	0.0000*	0.2550	0.0000*
Private	– 1.8440	0.0000*	– 1.8412	0.0000*
Cumulative monthly precipitation (mm)			– 0.0004	0.0117*
Maximum 5-day precipitation for a month (mm)			0.0005	0.1175
4-month lag monthly total precipitation (mm)			– 0.0002	0.3706
9-month lag monthly total precipitation (mm)			– 0.00006	0.7150
1-month lag monthly 5-day max (mm)			0.0006	0.0965

$M_0$ is the model without precipitation variables, whilst $M_1$ includes monthly cumulative precipitation, five-day maximum cumulative precipitation, their interactions, and lagged terms of these variables. Starred p-values indicate significance at $p < 0.05$. Categorical data was sourced from Malawi’s Harmonized Health Facility Assessment^39^, with precipitation indices calculated from the ERA5 reanalysis data^29^.

## Projecting forward: future disruptions under different climate scenarios

We considered three divergent future scenarios, based on the Shared Socioeconomic Pathways (SSP) framework^40^ (which describes variant future climate scenarios depending on different assumptions of global development and resource use section “Methods”): SSP1-2.6, SSP2-4.5, SSP5-8.5. These correspond to warming temperatures, globally, of approximately 1.7 $^\circ \hbox {C}$, 2 $^\circ \hbox {C}$, and 2.4 $^\circ \hbox {C}$, respectively, by 2060, our horizon of interest. Using projections on future pregnancies from the Thanzi La Onse (tlomodel.org) model, a Malawi-specific, individual-based, multi-disease and healthcare system model (section see “Methods”)^38,41^, we found that up to a quarter of a million pregnancies could be impacted by precipitation by 2060.

**Fig. 3:**
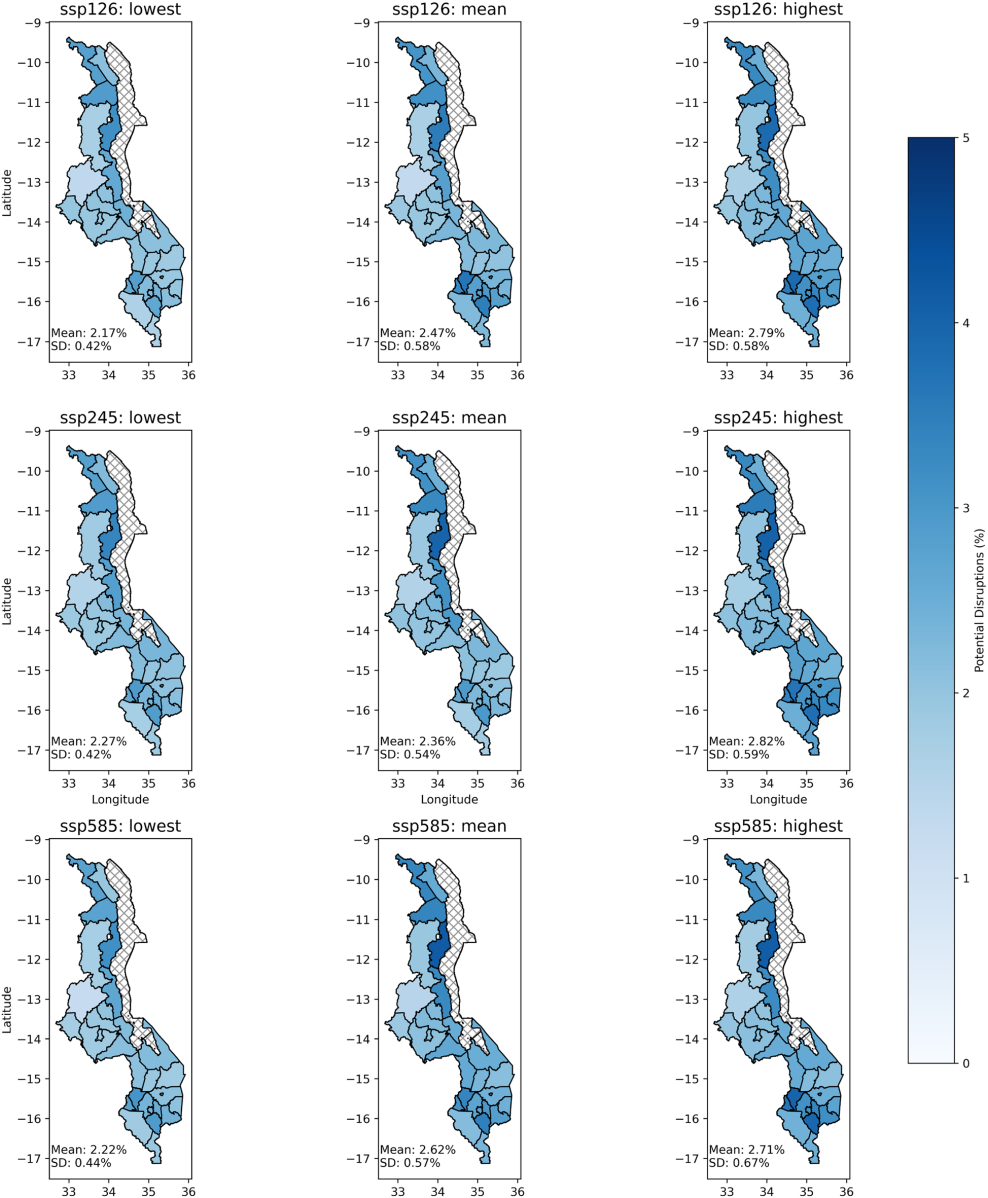
Projected percentage of ANC services potentially disrupted by precipitation events between 2025 and 2060. Maps were created by the first author in Python using Shapely 2.1.1^36^.

Irrespective of the scenario and model used, districts directly bordering Lake Malawi and in the south suffered the most disruption (Fig. 3), areas which have been highlighted as particularly vulnerable to future flooding and landslides^7^.

The cumulative magnitude of disruptions is similar across all scenarios (Table 2). This may be due to the effects of disparate effects during the wet and dry seasons^10,15^, and non-linear impact of warming on precipitation. Though extreme precipitation events are likely to increase, so too are periods of little to no rainfall^7^. Given that our model indicates that increase in monthly precipitation is associated with a decrease in ANC services, extended dry spells could mean periods of little transport or road disruptions. That is not to say that drought will have positive, or even neutral, effects on ANC utilisation, but they would need to be accounted for explicitly in a model.

What is notable, however, is that in the more extreme climate scenarios, a higher percentage of disruptions are due to extreme precipitation events (318 mm over one month, which is the 90th percentile of historical rainfall between 2011-2024; Table 2) compared to the “moderate” SPP1-2.6. Even when the overall proportion of pregnancies affected by precipitation remains constant between scenarios, extreme events cause the most disruption in SSP5-8.5.

**Table 2:** Cumulative disruption in ANC visits between in the “historical” (2012–2024), counterfactual, and 2025–2060 scenarios, nationally.

Scenario	Model type	Disruptions due to precipitation (% ANC services missed)	Disruptions due to extreme precipitation (% ANC services missed)
Historical	—	65,703 (2.52)	23,600 (35.92)
1941 - 1953 counterfactual	—	414 (0.016)	– 155 (37.53)
ssp126	Lowest	195,177 (1.86)	59,910 (32.63)
ssp126	Mean	230,889(2.29)	83,263 (34.96)
ssp126	Highest	249,790 (2.58)	89,950 (33.86)
ssp245	Lowest	212,723 (2.10)	76,641 (37.23)
ssp245	Mean	216,686 (2.16)	80,917 (37.30)
ssp245	Highest	248,516(2.61)	76,346 (30.15)
ssp585	Lowest	208,949 (1.93)	69,282 (34.12)
ssp585	Mean	240,781 (2.42)	96,187 (38.67)
ssp585	Highest	253,068 (2.55)	87,944 (33.71)

Extreme precipitation is based on the 90th percentile of the historical rainfall between 2011 and 2024 (318 mm per month).

Even given this difference, the general similarity in projections indicate that, even if emissions are reduced and the SSP1-2.6 pathway is achieved, Malawi can expect precipitation-mediated disruptions to healthcare access, particularly under extreme weather events.

## A different baseline: precipitation trends from 1940 to 1950

It is evident from our analysis that the patterns of ANC disruption between 2012 and 2024 are largely carried forward to 2060, irrespective of variations SSP. This may be attributable to the existing effects of climate change, which have already induced profound changes in Malawi’s climate^7^. Therefore, to understand the potential effect of anthropogenic climate change on ANC disruptions, we must use precipitation data from an earlier period.

We used ERA5 reanalysis precipitation data for 1941–1953 as a counterfactual data for our $M_1$ model for 2011-2024. Under this scenario, we find a much reduced impact of precipitation on the disruption of ANC visits: only 414 (0.016%) of pregnancies suffered any disruption, which is 99.3% fewer than the “factual” (“historical”) scenario. The pattern of geographical disruption was preserved.

## Discussion

Healthcare resilience under a changing climate is a topic of emerging concern^10,42^. To date, little work has been done to understand what risks extreme weather events pose to healthcare access, particularly in low- and middle-income countries. Here, we show that ANC service delivery in Malawi is adversely affected by precipitation events, particularly extreme events.

We estimate that, between 2012 and 2024, around 2.5% of pregnancies in Malawi were affected by precipitation-mediated disruptions to ANC care (assuming that for each live birth, there have been 4.4 ANC appointments per pregnant women). In the coming decades up to 2060, between 1.9 and 2.6% of ANC services will be disrupted, depending on the SSP scenario, translating into approximately 195,000 and 253,000 affected pregnancies. The cumulative projected disruptions, therefore, largely match historical patterns, potentially due to the impact of existing climate change^40^, the impacts of which are already being felt, and will continue to be felt for decades. However, using precipitation data from 1941-1953, there is a marked reduction in the number of disruptions, with fewer than 0.02% of the pregnancies affected, suggesting what climate change has occurred between 1953 and 2024 has negatively impacted ANC service utilisation in Malawi.

Across all scenarios, the broad similarity in national trends between scenarios belies regional and temporal variation. Whilst the mean cumulative potential disruption by district was 2.5% (standard deviation of 0.64%), districts that are in the South or bordering Lake Malawi may experience disruptions of up to 1 in 20 annual appointments, approximately 33% worse than historical patterns.

An additional potential reason for the lack of inter-scenario variability is because, though higher temperatures increase the likelihood of extreme precipitation events, they do so too for drought. We have treated precipitation as a continuous variable, with no minimum threshold for disruption, thus allowing for the possibility that whilst there will be more extreme events that could cause major disruptions, if “normal” precipitation events will become less common, these lower-level disruptions will disappear. Though extended periods of low precipitation, or even drought, are implicitly included in our analyses at a monthly resolution, the further inclusion of drought metrics as defined by the ETCCDI would provide further insight into this relationship.

In other “impact attribution” studies, where the consequences of extreme weather events are probabilistically associated with climate change^43^, the typical baseline “pre-anthropogenic climate change” climate is taken as 1850-1900, before the onset of rapid industrialisation and use of fossil fuels. However, in our ERA5 reanalysis dataset, the earliest available data are from 1940. Despite this limitation, data from this period still precede the observed rise in extreme precipitation events^10^, making it a reasonable baseline for our analysis.

Previous work in this area is limited, but estimates of ANC provision across Malawi in the six weeks preceding and succeeding Cyclone Freddy in a subset of clinics in the South West of Malawi showed a 60% reduction in ANC usage^16^. Though our analysis works at a different resolution, and predicts based on historical trends rather than the immediate pre- and post-event periods, we note that for February 2023 there was an estimated mean disruption to ANC services of approximately 5% across the entire region, with facility-level disruptions of up to 19%.

It is possible that we are under-estimating the level of disruptions. The monthly resolution of our data means that if an appointment is delayed, but occurs later in the same calendar month, it is not recorded as disrupted. Additionally, though we do account for the Euclidean distance to the closest clinic in our models, women may travel to further clinics, or preferentially travel to those that are accessible by paved roads or other transport links. These compensatory mechanisms were not possible to discern with the data we had available to us. Conversely, by not accounting for potential improvements in infrastructure, we could over-estimate the negative impact of precipitation on healthcare provision and utilisation.

This work only considers the simple relationship between precipitation and utilisation of ANC services. Whilst it is possible that a large part of the effect of precipitation is mediated by reduced accessibility of healthcare facilities, we have not included it explicitly via models of flooding and/or road networks, which - based on other regional studies - are likely to be mediators of the relationship that we have quantified.

Data scarcity meant that only a subset of all healthcare facilities in Malawi were included in our study (26.2% of all facilities expected to report on ANC services by DHIS2), meaning that smaller and potentially more vulnerable sites have been omitted. Furthermore, in resource-limited environments, if an extreme weather event, routine ANC services may be de-prioritised in favour of more urgent needs. Neither of these effects are accounted for in our model.

It is also possible that the disruptions we observe are because precipitation and associated flooding will limit the provision of healthcare. Just as patients may have difficulty accessing clinics, so too may healthcare providers, and supply chains disruptions may result in consumable stock-outs. As happened in Cyclone Freddy, healthcare clinics may be damaged and/or destroyed, limiting or eliminating the provision of care.

Even with these limitations, our analysis demonstrates that the upward trend in precipitation levels, particularly when linked to extreme events, will have negative consequences for the utilisation of ANC services in Malawi. Given the link between access to these services and improved maternal and neonatal outcomes^25^, such disruptions could have severe consequences for health if left unaddressed.

What is absent from our analysis is any inclusion of the long-term health outcomes of these disruptions in ANC provision. While many cases will be simply be postponed, it is likely that some will be missed entirely. This is particularly pertinent in Malawi, where such barriers to care could exacerbate and already-high maternal and neonatal mortality rate. This is also likely to have an inequitable effect: a wide range of socio-economic factors have been associated with the uptake of ANC in Malawi^20^, many of which are also linked to increased climate vulnerability^40,44^.

## Conclusion

Our analysis highlights that extreme precipitation events already disrupt ANC service delivery in Malawi and, even under varying future climates, will continue to do so. We find that precipitation-induced disruptions could affect hundreds of thousands of pregnancies in Malawi, with potential consequences for maternal and neonatal health outcomes. The low-elevation southern districts and areas bordering Lake Malawi will experience worse impacts, indicating that accessibility due to flooding may be a mediating pathway for these disruptions. These results highlight the need for health system resilience to include improvements to infrastructure, and to consider climate risk in future health system planning.

## Methods

### ANC data

We obtained data on the monthly number of antenatal care (AntenatalTotal) visits from Malawi’s District Health Information Systems (DHIS2)^28^. These data are available monthly, setting the temporal resolution of our analysis. After cross-referencing with clinics for which we had data available from Malawi’s Malawi’s Harmonized Health Facility Assessment^39^, and omitting clinics where >90% of observations were missing, we obtained ANC visit data for 199 healthcare facilities (26.2 % of the rural and urban hospitals, healthcare clinics, and health centres expected to report data to DHIS2) from 2011 and 2024. Excluding missing data in the remaining clinics, we have 21,133 observations for this time period. For details on how we dealt with missing data, see Appendix “Missing data from the electronic health records”.

### Climate data

We obtained daily historical precipitation levels in Malawi from ERA5, a gridded reanalysis product produced by European Centre for Medium-Range Weather Forecasts (ECMWF)^29^. We obtained data from 2011 to 2024, and used it to calculate the cumulative monthly precipitation for each month in that period and 1,2,3,4, and 9-month lag in precipitation. Additionally, we calculated the 5-day maximum cumulative precipitation for each month^35^, and included the same lag periods as the monthly data. The square of the cumulative monthly and 5-day max indices were included, alongside the interaction between them.

For estimates of future precipitation, we used downscaled Coupled Model Intercomparison Project phase 6 from the Carbon Impact Lab^30^. These data are at a 1/4-degree regular global grid and use ERA5 as a reference dataset. We examined three Shared Socio-Economic Pathways (SSPs), which represent different scenarios for the future climate depending on societal, demographic, and economic trends^40^. These scenarios were SSP1-1.9, which would result from sustainable growth and increased societal equality; SSP2-4.5, the “middle of the road” which broadly follows historical patterns across all factors of interest, and SSP5-5.8, which results from increased fossil-fuel driven development.

To avoid averaging out extremes in precipitation events, for each of the scenarios, we chose the model projections with the lowest, mean, and highest mean monthly prediction over all grid squares in the 35-year period of interest (Table 3).Using the geographic co-ordinates for each facility, we used the KDTree algorithm^34^ to assign each facility to the appropriate ERA5 grid, linking the facility and precipitation data.

**Table 3:** Downscaled CMIP6 models comprising the model ensemble in our analysis.

Scenario	Lowest	Median	Highest
SSP1-1.9	HadGEM3-GC31-LL	MPI-ESM1-2-LR	INM-CM5-0
SSP2-4.5	HadGEM3-GC31-LL	UKESM1-0-LL	INM-CM5-0
SSP5-8.5	HadGEM3-GC31-LL	EC-Earth3-CC	CMCC-CM2-SR5

By the time of our historic analysis (2011–2024), Malawi’s climate was already changing. Therefore, to estimate what the impact of precipitation would be if there were minimal effects of climate change, we obtained ERA5 precipitation data for 1941–1953. We calculated the same metrics as in the main analysis.

### Covariate data

In our initial model fitting, we included the following covariates: the precipitation indices (cumulative monthly rainfall (mm), 5-day maximum monthly rainfall (mm), the 1-,2-,3-,4-,9-month lag in these metrics (mm), and their squares (mm$^2$), cubes (mm$^3$) and products (mm$^2$), the year and month of observation, the facility ID, its zone (Northern, Central East, Central West, South East, South West), type (Dispensary, Health Centre, Clinic, Rural/Community Hospital, District Hospital), district, and owner (Government, Christian Health Association of Malawi, NGO, or Private), whether it was urban or rural, the Euclidean distance to the closest facility (m), and its altitude (m).

A covariance matrix indicated a high level of correlation between the facility owner and the facility type; we therefore removed the latter from the predictions. Similarly, zone was highly correlated with the district, so we removed the latter.

### Projections of pregnancy

We used the TLO model (tlomodel.org,^45^) to estimate the number of pregnancies for the years 2011–2060. TLO is an individual-based, all-disease, whole health-system model specific to Malawi, though at present, these projections are made in the absence of information on climate change. Therefore, the birth projections are constant across all future scenarios. However, the model does account for changes in the socio-economic profile of the Malawian population over time. Of particular relevance for our study, given our hypotheses concerning accessibility of clinics, that there is an increase in the urban population.

Additionally, in order to generate projections on births, we use an idealised scenario in which there are no limitations on the availability of or access to healthcare (i.e., there are no limitations on the availability of consumables and equipment, healthcare workers can operate to their fullest capacity, and those who need medical attention seek it)^38,41^.

### Missing data from the electronic health records

There appeared to be a pattern of non-randomness in the DHIS2 entries around the time of Cyclone Freddy in Malawi (February-March 2023). In particular, when news reports^9^ confirmed that a Phalombe Clinic was closed due to cyclone damage, entries in DHIS2 were missing, rather than “0” (indicating that no services were delivered). Yet for Nkhulabme Health Centre, which was also closed due to damage, “0” services were returned to DHIS2. A third clinic, which is in close proximity with these two clinics - the Thumbwe Health Centre - also reported missing data, suggesting it too was affected by the cyclone, though no news reports could confirm this.

To account for this, where secondary sources could confirm the closure or otherwise of a clinic, the missing data were replaced with a “0” (indicating no services were delivered). However, where such sources could not be confirmed, we assumed that the lack of data was due to random non-reporting, and not associated with weather.

Missing data on altitude were imputed based on the mean altitude of all clinics.

Likoma had no clinics with the requisite covariate data, so was omitted from the analyses.

### Analysis

We constructed two models to predict the future number of antenatal care (ANC) services delivered each month in each clinic: one with no information on precipitation, and one with. Both of these were linear regression models, using logged counts of ANC services for each month as our outcome variable.

We used backward stepwise selection to choose the best model based on Akaike information criterion (AIC), resulting in the following:

Model $M_0$, without precipitation indices (AIC = 63501):

1 $$\begin{aligned} \begin{aligned} \log (y_{0_{i,t}} + \epsilon ) = \\ &\beta _0 + \beta _1 X_{\text {Year}} + \beta _2 X_{\text {Month}} + \beta _3 X_{\text {Urban}} + \beta _4 X_{\text {Central West}} + \beta _5 X_{\text {Northern}} \\ &+ \beta _6 X_{\text {South Eastern}} + \beta _7 X_{\text {South Western}} + \beta _8 X_{\text {Government}} + \beta _9 X_{\text {Private}} \\ &+ \beta _{10} X_{\text {Altitude}} + \beta _{11} X_{\text {Distance to closest clinic}} \end{aligned} \end{aligned}$$

where $\epsilon$ = 1 to allow for the calculation of logs when the number of ANC services delivered are 0.

The model $M_1$ with precipitation indices is as follows ($\Delta AIC = -6$):

2 $$\begin{aligned} \begin{aligned} \log (y_{1_{i,t}} + \epsilon ) =\\ &\beta _0 + \beta _{1} X_{\text {Cumulative Precipitation}, t} \\ &+ \beta _{2} X_{\text {5-day Maximum Precipitation}, t} + \beta _{3} X_{\text {Cumulative Precipitation}, t-4} \\ &+ \beta _{4} X_{\text {5-day Maximum Precipitation}, t-1} + \beta _{5} X_{\text {Cumulative Precipitation}, t-9} \\ &+ \beta _{6} X_{\text {Year}} + \beta _{7} X_{\text {Month}} \\ &+ \beta _{8} X_{\text {Urban}} + \beta _{9} X_{\text {Central West}} \\ &+ \beta _{10} X_{\text {Northern}} + \beta _{11} X_{\text {South East}} + \beta _{12} X_{\text {Government}} + \beta _{13} X_{\text {Private}} \\ &+ \beta _{14} X_{\text {Altitude}} + \beta _{15} X_{\text {Distance to closest clinic}} \end{aligned} \end{aligned}$$

In both models, categorical variables (facility ownership (Government, Non-Governmental Organisation (NGO), or Private), Region (Northern, Central Western, Central Eastern, South Eastern, South Western) and location (Urban or Rural)) were one-hot encoded to control for their effects.

Coefficients and p-values for these models are shown in Tables 1–3. Both models had a high adjusted $R^2$ ($> 0.95$).

We considered the difference in the predictions of these two models to be the number of ANC services that were disrupted due to precipitation for month *i* in year *j* (Eq. 3, where the 0 subscript indicates no precipitation indices, and 1 their inclusion)^15^. We reason that the omission of precipitation indices will give us the expected number of ANC services delivered assuming that precipitation has no effect. Therefore, if inclusion of the indices changes the predicted ANC services delivered, we attribute that difference to precipitation. A likelihood ratio test indicated that model $M_1$ was better at predicting the data than the $M_0$, precipitation-free model (LR: 14.0 on 4 degrees of freedom, p-value = 0.007).

As we are primarily interested in the negative disruptions of precipitation events, in presenting our results we will only consider months where the “precipitation” model predicts a deficit of ANC services delivered. Given the monthly resolution of our data, we were not able to capture any appointments that were postponed by a few days but still fell within the recording month of interest: only ones that were missed entirely, or shifted to the next month (inflating those figures) could be captured. Our inclusion of lagged precipitation metrics helps to account for the latter effect.

3 $$\begin{aligned} \Delta ANC_{i, j} = y_{0, i, j} - y_{1, i, j} \end{aligned}$$

## Data Availability

The datasets analyzed during the current study are available in the ECMWF Copernicus repository (https://cds.climate.copernicus.eu/) and DHIS2 (https://dhis2.org/). Data on clinic locations and co-variables are available from the corresponding author (rm916@ic.ac.uk) upon reasonable request.
